# Ciprofloxacin Improves the Stemness of Human Dermal Papilla Cells

**DOI:** 10.1155/2016/5831276

**Published:** 2015-11-16

**Authors:** Chayanin Kiratipaiboon, Parkpoom Tengamnuay, Pithi Chanvorachote

**Affiliations:** ^1^Pharmaceutical Technology (International) Program, Faculty of Pharmaceutical Sciences, Chulalongkorn University, Bangkok 10330, Thailand; ^2^Department of Pharmaceutics and Industrial Pharmacy, Faculty of Pharmaceutical Sciences, Chulalongkorn University, Bangkok 10330, Thailand; ^3^Department of Pharmacology and Physiology, Faculty of Pharmaceutical Sciences, Chulalongkorn University, Bangkok 10330, Thailand; ^4^Cell-Based Drug and Health Product Development Research Unit, Faculty of Pharmaceutical Sciences, Chulalongkorn University, Bangkok 10330, Thailand

## Abstract

Improvement in the expansion method of adult stem cells may augment their use in regenerative therapy. Using human dermal papilla cell line as well as primary dermal papilla cells as model systems, the present study demonstrated that ciprofloxacin treatment could prevent the loss of stemness during culture. Clonogenicity and stem cell markers of dermal papilla cells were shown to gradually decrease in the culture in a time-dependent manner. Treatment of the cells with nontoxic concentrations of ciprofloxacin could maintain both stem cell morphology and clonogenicity, as well as all stem cells markers. We found that ciprofloxacin exerted its effect through ATP-dependent tyrosine kinase/glycogen synthase kinase3*β* dependent mechanism which in turn upregulated *β*-catenin. Besides, ciprofloxacin was shown to induce epithelial-mesenchymal transition in DPCs as the transcription factors ZEB1 and Snail were significantly increased. Furthermore, the self-renewal proteins of Wnt/*β*-catenin pathway, namely, Nanog and Oct-4 were significantly upregulated in the ciprofloxacin-treated cells. The effects of ciprofloxacin in preserving stem cell features were confirmed in the primary dermal papilla cells directly obtained from human hair follicles. Together, these results revealed a novel application of ciprofloxacin for stem cell maintenance and provided the underlying mechanisms that are responsible for the stemness in dermal papilla cells.

## 1. Introduction

Based on the fact that dermal papilla cells (DPCs) interaction with epithelial stem cells can induce generation of new hair follicles [1–3], the cell therapy using DPCs has emerged as a potentially new approach for hair transplantation [4, 5]. DPCs have been intensively investigated to possess many advantages for cell therapy approaches. However, many studies also demonstrated the loss of their stemness and inductive activity during the* in vitro* passages [4, 6–8].

The hair follicle is composed of epithelial and mesenchymal compartments. DPCs, the major cell population existing in the mesenchymal compartments, are located at the base of the hair follicle and function as a signaling center in the hair follicle morphogenesis and growth cycle [9]. These cells instruct the epithelial stem cells through specific signals to proliferate and differentiate into multiple layers of the growing hair shaft [1–3]. Interestingly, DPCs have been characterized as multipotent stem cells and the stemness of such cells is tightly associated with the ability to induce hair follicle formation. With comprehensive knowledge of the stem cell biology, the evidence suggested that CD133, a protein marker of human stem cells, contributes to the hair inductive property of DPCs in transgenic mice [10, 11]. In addition, an ablation of stem cell-related transcription factors including Sox2 in DPCs leads to the impairment of the hair shaft outgrowth [12]. Although the molecular features that regulate stemness as well as hair inductive function in these specialized DPCs are still largely unknown, the Wnt/*β*-catenin signaling appears to lend strong support to hair follicle morphogenesis and regeneration [13–15]. Indeed, *β*-catenin was shown to regulate crucial signaling pathways in hair follicle formation in response to several stimuli, including fibroblast growth factor (FGF) and insulin-like growth factor (IGF) [13]. In transgenic mice model, the suppression of *β*-catenin in the DPCs resulted in the inhibition of hair follicle formation [13], as epithelial-mesenchymal transition (EMT) lately has been shown to play an important role in the stem cell behaviors and the activation of Wnt/*β*-catenin signaling was shown to activate the transition of the epithelial cells toward mesenchymal stem cells. Together with the concept that transcription factors presenting in the cell undergoing EMT like Snail were found to be important for the accomplishment of stem cell functions [16–18], it is likely that these *β*-catenin and EMT could impact the stemness and stem cell activity in the DPCs.

Ciprofloxacin (CIP) has been used as an antibiotic prophylaxis for the prevention of bacterial infection in patients receiving stem cell transplant and in stem cell research [19, 20]. Also, this considerably safe drug is widely used in the treatment of certain infection in cell culture [21]. So far, the molecular basis of CIP on human cell biology has not been fully investigated, especially in the area of stem cell research. The present study therefore aimed to elucidate the possible role of CIP for its possible effect on the stemness of DPCs using human dermal papilla cell line and primary human dermal papilla cells as models.

## 2. Material and Methods 

### 2.1. Cells and Reagents

Immortalized dermal papilla cells (DPCs) were obtained from Applied Biological Materials Inc. (Richmond, BC, Canada). The cells were cultured in Prigrow III medium (Richmond, BC, Canada) supplemented with 10% fetal bovine serum (FBS) and 100 units/mL of penicillin/streptomycin (Life technologies, MD, USA) at 37°C in a 5% CO_2_ atmosphere. For primary human DPCs, they were obtained from PromoCell (Heidelberg, Germany). The cells were cultured in medium containing bovine pituitary extract 4 *μ*L/mL, fetal calf serum 0.05 mL/mL, basic fibroblast growth factor 1 ng/mL, recombinant human insulin 5 *μ*g/Ml and phenol red 0.62 ng/mL from PromoCell (Heidelberg, Germany), and 100 units/mL of penicillin/streptomycin at 37°C in a 5% CO_2_ atmosphere. Ciprofloxacin (CIP) and dimethylsulfoxide (DMSO) were purchased from Sigma (St. Louis, MO, USA). Hoechst 33342 and propidium iodide (PI) were obtained from Molecular Probes Inc. (Eugene, OR, USA). 3-(4,5-Dimethylthiazol-2-yl)-2,5-diphenyltetrazolium bromide (MTT) and Alexa Fluor 488/594 conjugated secondary antibody were from Invitrogen (Carlsbad, CA, USA). Rabbit monoclonal antibodies for integrin*β*1, phosphorylated ATP-dependent tyrosine kinase (Akt, Ser 473), Akt, phosphorylated glycogen synthase kinase3*β* (GSK3*β*, Ser 9), GSK3*β*, ZEB1, Nanog, Oct-4, Slug, Snail, Vimentin, N-cadherin, phosphorylated extracellular signal-regulated kinase (Erk), Erk, *β*-actin, and peroxidase conjugated anti-rabbit IgG were obtained from Cell Signaling (Denver, MA, USA). Rabbit CD133 antibody was bought from Cell Applications Inc. (San Diego, CA, USA). Rabbit procollagen type I antibody, goat aldehyde dehydrogenase 1A1 antibody (ALDH1A1), and peroxidase conjugated anti-goat IgG were obtained from Santa Cruz Biotechnology Inc. (Dallas, Texas, USA). Immobilon Western Chemiluminescent HRP substrate was from Millipore Corp. (Billerica, MA) and Thermo Fisher Scientific Inc. (Rockford, IL).

### 2.2. Cell Viability Assay

MTT viability assay was used to evaluate cell viability. Cells were seeded at a density of 1 × 10^4^ cells/well and cultivated for 12 h in 96-well plate. Afterward, the cells were incubated with various concentrations of CIP (0–10 *μ*g/mL) for 24 h. The cells were then incubated with 5 mg/mL MTT for 4 h at 37°C. Then, the supernatant was removed and replaced with 100 *μ*L of DMSO to dissolve the formazan crystal. The intensity of MTT product was measured at 570 nm using a microplate reader (Anthos, Durham, NC). Cell viability was calculated by the following formula and presented as a percentage to untreated control value

(1) $$\begin{array}{c} Cell\;viability\left(\%\right)=\frac{A570\;of\;treatment}{A570\;of\;control}\times 100. \end{array}$$

### 2.3. Nuclear Staining Assay

Hoechst 33342 and PI costaining was used to detect apoptotic and necrosis cell death. Cells were seeded at a density of 1 × 10^4^ cells/well and cultivated for 12 h. Subsequently, the cells were treated with various concentrations of CIP (0–10 *μ*g/mL) for further 24 h. After treatments, the cells were stained with 10 *μ*M of Hoechst and 5 *μ*g/mL of PI for 30 min at 37°C and visualized by fluorescence microscope (Olympus IX 51 with DP70, Olympus America Inc., Center valley, PA).

### 2.4. Cell Morphology and Aggregation Behavior Evaluation

DP cells were seeded at a density of 6 × 10^3^ cells/well onto 24-well plate and incubated for 12 h. The cells were treated with various concentrations of CIP (0–10 *μ*g/mL) for 72 h, and cell morphology was observed at 0, 24, 48, and 72 h. The aggregation behavior of the cells was determined at 72 h. Morphology and aggregation behaviors of cells were photographed by a phase-contrast microscope (Olympus IX51 with DP70, Olympus America Inc., Center valley, PA).

### 2.5. Cell Cycle Analysis

Cells were seeded at a density of 3 × 10^4^ cells/well onto 6-well plate and incubated overnight. The cells were cultured in the presence or absence of CIP (10 *μ*g/mL) for 72 h. After indicated treatment, the cells were incubated in the absence of growth factors for 24 h. The cells were then incubated with complete media for 12 h, trypsinized and fixed with 70% absolute ethanol at −20°C overnight. The cells were washed with cold PBS and incubated in PI solution containing 0.1% Triton-X, 1 *μ*g/mL RNase, and 1 mg/mL propidium iodide at 37°C for 30 min. The cells at the early passages (passages 2-3) without serum-starvation were used as an untreated control at 0 h. DNA in whole cells was stained with PI, and cell cycle profile was analyzed using flow cytometry (FACSort, Becton Dickinson, Rutherford, NJ, USA).

### 2.6. Immunofluorescence

Cells were seeded at a density of 3 × 10^4^ cells/well onto coverslips in 6-well plate and incubated overnight. The cells were cultured in the presence or absence of ciprofloxacin (10 *μ*g/mL) for 72 h. The cells at the early passages were used as an untreated control at 0 h. The coverslips were fixed with 4% paraformaldehyde for 20 min and permeabilized with 0.1% Triton-X for 10 min at room temperature. Thereafter, the coverslips were incubated with 3% bovine serum albumin (BSA) for 30 min at room temperature to prevent nonspecific binding. The coverslips were washed and incubated with CD133 or procollagen type I rabbit monoclonal antibodies at 1 : 100 dilution overnight at 4°C. After primary antibody incubation, the coverslips were washed with PBS and subsequently incubated with Alexa Fluor 488 or 594 conjugated secondary antibodies for 1 h at room temperature. Samples were examined with Confocal Laser Scanning Microscopy (Zeiss LSM 510) to analyze expression of CD133 and procollagen type I.

### 2.7. Western Blot Analysis

Cells were seeded at a density of 5 × 10^4^ cells/well onto 6-well plate for 12 h and cultured in the presence of various concentrations of CIP (2.5–10 *μ*g/mL) for 72 h. After washing the cells with PBS, cell lysates were prepared by incubating the cells in ice-cold lysis buffer containing 20 mM Tris·HCl (pH 7.5), 0.5% Triton X, 10% glycerol, 150 mM sodium chloride, 50 mM sodium fluoride, 1 mM sodium orthovanadate, 1 mM phenylmethylsulfonyl fluoride, and commercial protease inhibitor cocktail (Roche Molecular Biochemicals) for 45 min on ice. Subsequently, cell lysates were collected and determined for protein content by the Bradford method (Bio-Rad, Hercules, CA). Equal amounts of proteins of each sample (50 *μ*g) were boiled in Laemmli loading buffer at 95°C for 5 min. The proteins were subsequently loaded on 10% SDS-polyacrylamide electrophoresis. After separation, proteins were transferred onto 0.45 *μ*m nitrocellulose membranes (Bio-Rad). Following blocking with 5% nonfat milk in TBST [25 mM Tris·HCl (pH 7.5), 0.05% Tween-20, and 125 mM NaCl] for 2 h, membranes were then incubated with appropriate primary antibodies for 10 h at 4°C. Membranes were washed three times with TBST for 15 min and incubated with horseradish peroxidase-coupled secondary antibodies for 1 h at room temperature. The immune complexes were detected with Chemiluminescence substrate (SuperSignal West Pico, Pierce, Rockford, IL) and quantified using analyst/PC densitometry software (Bio-Rad).

### 2.8. Statistical Analysis

Data were obtained from at least four independent experiments and presented as means ± standard deviation (SD). Statistical analysis was performed using one-way ANOVA with post hoc test at a significance level (*α*) of 0.05. These analyses were performed using SPSS Version 19 (SPSS Inc., Chicago, IL).

## 3. Results

### 3.1. Effect of CIP on Viability of DPCs

**Figure 1:**
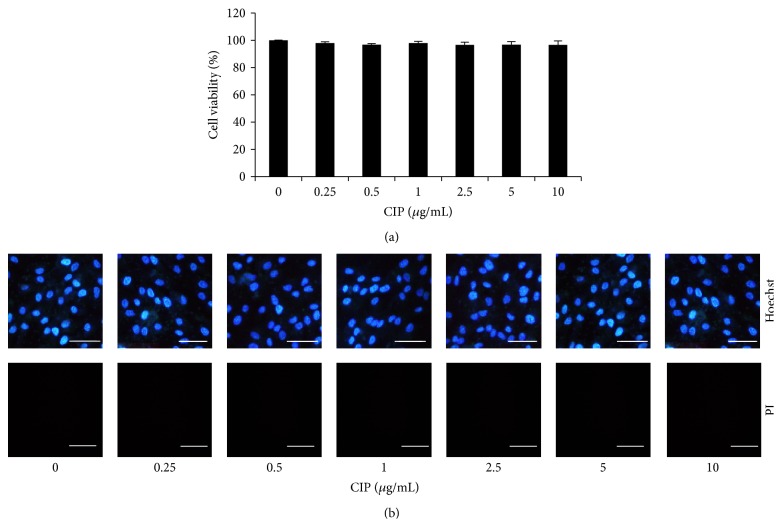
Cytotoxicity of CIP on DPCs. (a) Cells were treated with CIP (0–10 *μ*g/mL) for 24 h. Cytotoxicity was determined by MTT assay. (b) After indicated treatment for 24 h, mode of cell death was examined by Hoechst 33342/PI costaining assay. Scale bar is 100 *μ*m. The data represent the means of four independent samples ± SD.

To study the role of CIP on the stem cell property of DPCs, we first characterized cell viability and cell death response to CIP treatment in DPCs using MTT and Hoechst 33342/propidium iodide (PI) costaining assays. Treatment of the cells with CIP at the concentrations of 0–10 *μ*g/mL for 24 h caused no significant change in cell viability compared with control levels (Figure 1(a)). Consistent with the Hoechst/PI apoptosis assay, our results indicated that the treatment drug at such concentrations caused neither apoptosis nor necrosis detected by Hoechst and PI, respectively (Figure 1(b)). This information may help to clarify that the following effects of CIP on DPCs were not a consequence of cytotoxic effect or cell stress.

### 3.2. CIP Maintains Stem Cell-Like Characteristics in DPCs

**Figure 2:**
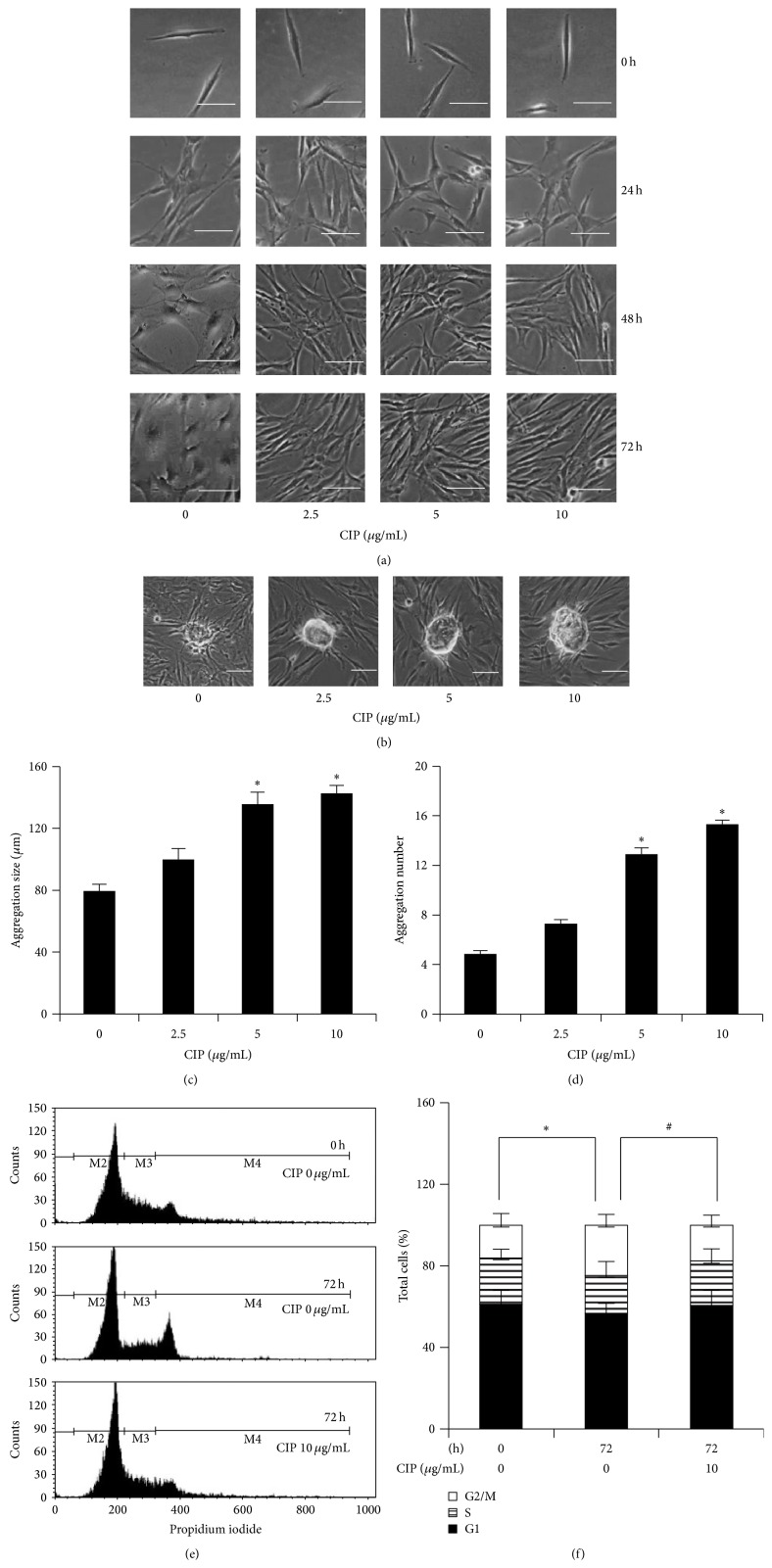
Effects of CIP on stem cell-like characteristics in DPCs. (a) Cells were treated with CIP (0–10 *μ*g/mL) for various times (0–72 h). After indicated treatment, morphology of DPCs was observed. Scale bar is 100 *μ*m. (b) Aggregation behavior of cells was determined after indicated treatment for 72 h. Scale bar is 100 *μ*m. ((c)-(d)) Aggregation size and aggregation number were determined by image analyzer. The data represent the means of four independent samples ± SD. ^*∗*^ *P* < 0.05 versus untreated control. ((e)-(f)) Cells were cultured in the presence or absence of CIP (10 *μ*g/mL) for 72 h and serum-starved for 24 h. After serum-starvation, cells were incubated with complete media for 12 h. The cells at the early passages (passages 2-3) without serum-starvation were also used as an untreated control at 0 h. Cell cycle distribution was determined by PI staining and flow cytometry. The data represent the means of four independent samples ± SD. ^*∗*^ *P* < 0.05 versus untreated control at 0 h; ^#^ *P* < 0.05 versus untreated control at 72 h.

DPCs have been reported to function as multipotent stem cells and the stemness of DPCs was linked to their ability to induce hair follicles [10–12]. However, DPCs lose their hair follicle inductive ability during culture [4, 6–8]. We found that, after culturing the DPCs for 5 days, the shape and appearance of DPCs are spontaneously altered toward fibroblast-like morphology. The primitive DPCs usually appearing as spindle-shaped cells changed to flat multipolar cells with elongated shapes (Figure 2(a)). Besides, the DPCs at the beginning showed an aggregative growth pattern in culture and such pattern was lost during the extended time of culturing. In order to test whether CIP affects the change in morphology of these DPCs, the cells were treated with CIP at the concentrations of 0–10 *μ*g/mL for 0–72 h, and morphology of the cells as well as aggregative pattern was determined. Figure 2(a) shows that most of untreated control cells exhibited fibroblast-like morphology at 48 and 72 h. Meanwhile, the morphology of CIP treated cells remained unaltered (Figure 2(a)). Because the hair follicle inductive property of the DPCs has been shown to relate with their aggregate behaviors [22], we further investigated the effect of CIP treatments on the aggregative growth pattern of the cells. The DPCs at early passages (passages 2-3) were cultured in the presence or absence of CIP for 72 h and the aggregate size and number were determined. Figures 2(b), 2(c), and 2(d) show that CIP at the concentration of 5 and 10 *μ*g/mL significantly increased the size as well as the number of cell aggregation in comparison to those of untreated control at 72 h.

As mesenchymal cells have been shown to be slow-cycling cells, we next investigated the effect of CIP on the proliferation and the cell cycle distribution of DPCs. The DPCs were cultured in the presence or absence of CIP for 72 h and subjected to cell cycle evaluation. The cells were incubated in the absence of growth factors for 24 h. Then, the cells were incubated with complete media for 12 h and the cell cycle progression was analyzed by PI and flow cytometer. Also, the DPCs at the early passages without serum-starvation were used as a control. Figures 2(e) and 2(f) show that, at 12 h after the cells receive growth factors, the untreated control cells at 72 h proceeded to M phase of the cell cycle. Importantly, treatment of the cells with 10 *μ*g/mL CIP attenuated the cell cycle progression as the cells cannot enter to M phase. Further, the cell cycle distribution was quantified as described in Section 2.5. The results confirmed the above findings that the treatment of the cells with CIP significantly decreased the cell population in G2/M phase (Figures 2(e) and 2(f)).

### 3.3. CIP Prevents the Downregulation of Stem Cell Markers in DPCs

**Figure 3:**
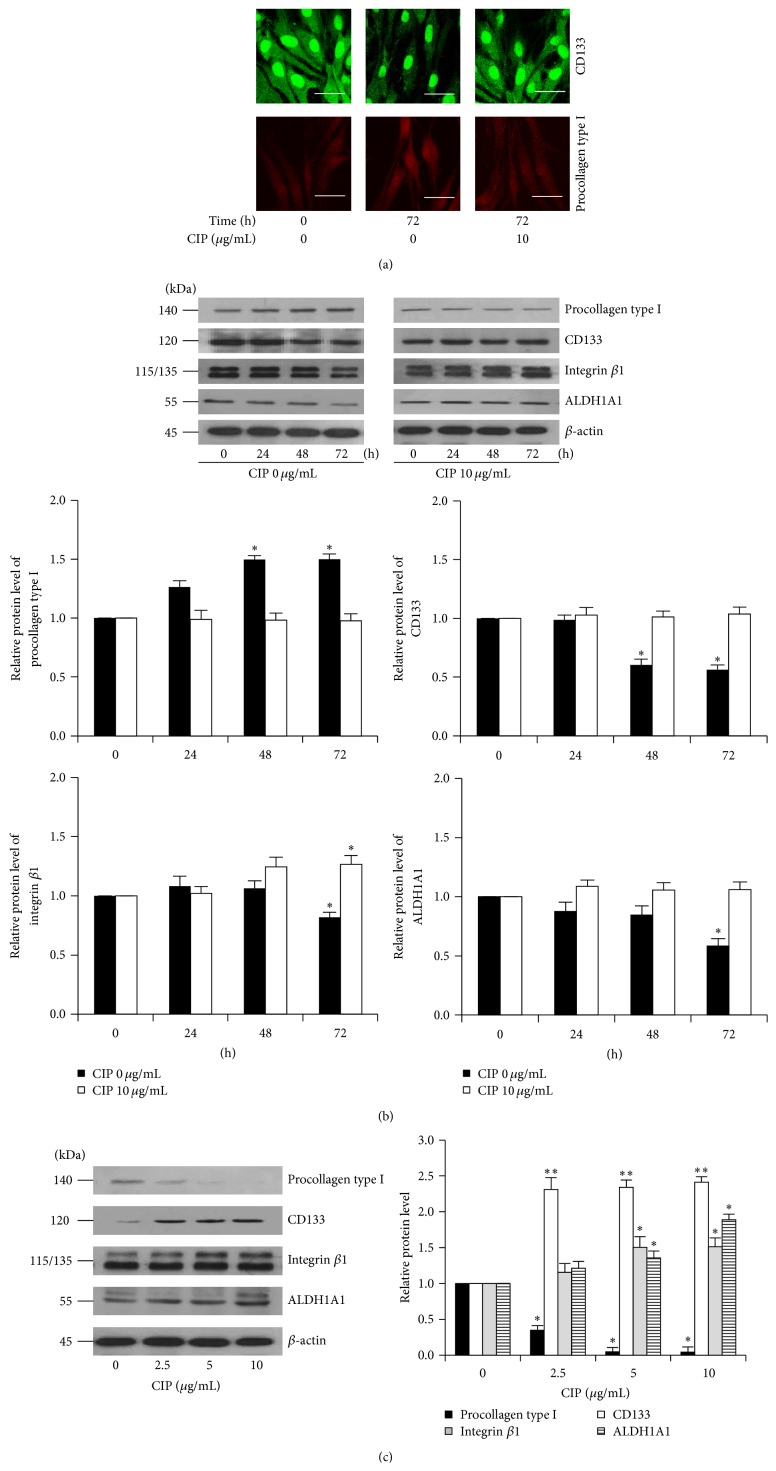
Effects of CIP on stem cell markers in DPCs. (a) Cells were cultured in the presence or absence of CIP (10 *μ*g/mL) for 72 h. The cells at the early passages were used as an untreated control at 0 h. Expression of CD133 and procollagen type I were analyzed by immunofluorescence staining. Scale bar is 50 *μ*m. (b) Time-dependent effects of CIP treatment on the expression of stem cell markers were determined. Cells were cultured in the presence or absence of CIP (10 *μ*g/mL) for 0–72 h. The levels of procollagen type I, CD133, integrin *β*1, and ALDH1A1 were determined by western blot analysis. Blots were reprobed with *β*-actin to confirm equal loading. The immunoblot signals were quantified by densitometry and the mean data from independent experiments were normalized to the results. The data represent the means of four independent samples ± SD. ^*∗*^ *P* < 0.05 versus untreated control at 0 h. (c) Cells were treated with CIP (0–10 *μ*g/mL) for 72 h. After indicated treatment, levels of procollagen type I, CD133, integrin *β*1, and ALDH1A1 were analyzed by western blot. *β*-actin was served as the loading control. The immunoblot signals were quantified by densitometry and the mean data from independent experiments were normalized to the results. The data represent the means of four independent samples ± SD. ^*∗*^ *P* < 0.05 and ^*∗∗*^ *P* < 0.01 versus untreated control.

Having shown that culture of the DPCs caused the spontaneous decline of stem cell-like phenotypes, we next clarify the mentioned conception by detecting stem cell markers in such cells. Because the CD133 and procollagen type I expressions have been recognized as the dermal papilla cell and fibroblast indicators, respectively [11, 23, 24], we analyzed the expression of such proteins in the DPCs treated with CIP at the concentrations of 10 *μ*g/mL for 72 h and the control cells. Immunocytochemistry showed that the expression of CD133 with CIP was suppressed in the DPCs cells after being cultivated for 72 h in comparison to that of control cells (DPCs at the early passages at 0 h, Figure 3(a)). Treatment of the cells with CIP could dramatically prevent such a loss of CD133 expression in the cells (Figure 3(a)). These results suggested that CIP preserve the stemness of DPCs during culture. Also, the expression level of fibroblast marker procollagen type I was significantly increased in the untreated DPCs cells at 72 h, whereas the increase in fibroblast marker could be prevented by CIP at the concentration of 10 *μ*g/mL (Figure 3(a)).

We further exploited the information to show that CIP prevents the loss of stemness in cultured DPCs. By utilizing CD133, integrin *β*1, and ALDH1A1 as dermal papilla markers and procollagen type I as a fibroblast marker, the cells cultured in the presence or absence of CIP were analyzed for the proteins by western blot analysis.  Figure 3(b) shows that the expression of procollagen type I was upregulated in a time-dependent manner, whereas treatment with CIP could prevent the increase of such a protein. As expected, all mesenchymal related proteins including CD133, integrin *β*1, and ALDH1A1 were gradually decreased in the untreated control cells in a time-dependent fashion and treatment of the cells with CIP inhibited the reduction of the protein markers (Figure 3(b)). We also performed the dose-dependent experiment to assure the effect of the drug on DPCs stemness. The results indicated that CIP increased the stem cell markers in these cells while it decreased fibroblast marker in a dose-dependent manner (Figure 3(c)).

### 3.4. CIP Activates Wnt/*β*-Catenin Signaling and Epithelial-Mesenchymal Transition (EMT) in DPCs

Activation of Wnt/*β*-catenin signaling was shown to play a critical role in stem cell maintenance [25–27] and hair regeneration [13–15]. In addition, the hair follicle inductive effect in cultured DPCs could be prolonged by exposing the cells to Wnt/*β*-catenin activator [14, 15]. Based on these data, it is promising that CIP may exert its positive role in stemness in DPCs through this pathway. The signaling proteins related to Wnt/*β*-catenin including activated Akt (phosphorylated Akt at Ser 473), total Akt, inactivated glycogen synthase kinase3*β* (phosphorylated GSK3*β* at Ser 9), parental GSK3*β*, and *β*-catenin were determined by western blot analysis.

**Figure 4:**
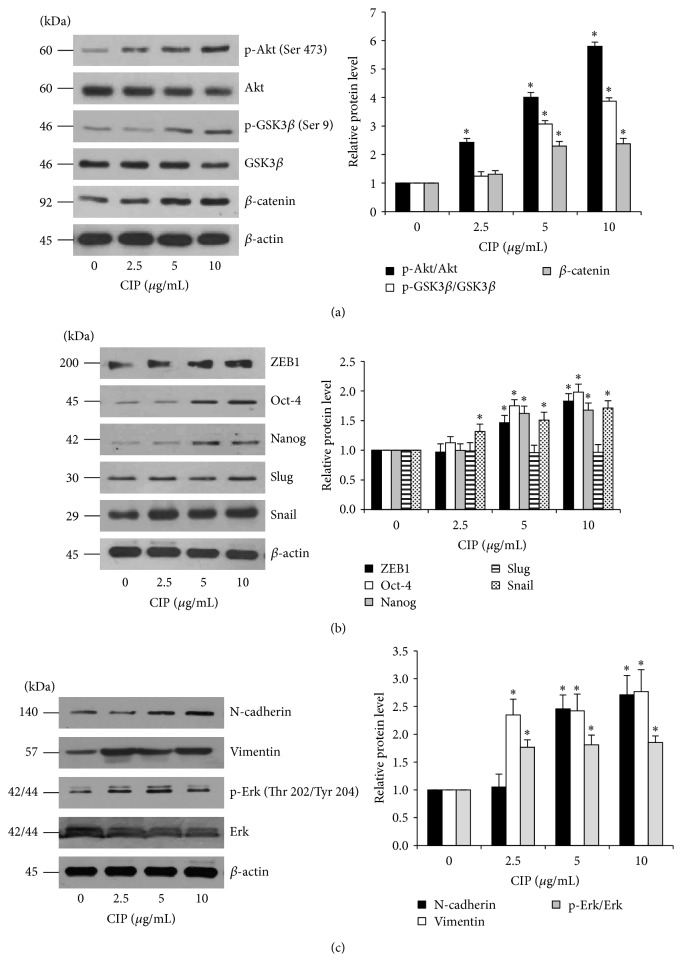
Effects of CIP on Wnt/*β*-catenin signaling, EMT, and self-renewal transcription factors in DPCs. (a) Cells were treated with CIP (1–10 *μ*g/mL) for 72 h. After indicated treatment, the levels of Wnt/*β*-catenin signaling (Akt, p-Akt (Ser 473), GSK3*β*, p-GSK3*β* (Ser 9), and *β*-catenin) were analyzed by western blot analysis. The immunoblot signals were quantified by densitometry and mean data from independent experiments were normalized to the results. (b) EMT and self-renewal transcription factors (ZEB1, Oct-4, Nanog, Slug, and Snail) were determined by western blot analysis. (c) Downstream targets of Snail including N-cadherin, vimentin, total Erk, and p-Erk (Thr 202/Tyr 204) were analyzed by western blot analysis. *β*-actin was used as the loading control. The western blot signals were quantified by densitometry and mean data from independent experiments were normalized to the results. The data represent the means of four independent samples ± SD. ^*∗*^ *P* < 0.05 versus untreated control.

The results showed that activated Akt was significantly enhanced by treatment of the cells with CIP at 2.5–10 *μ*g/mL in a dose-dependent manner (Figure 4(a)). Consequently, the downstream GSK3*β* was inactivated by the treatment of CIP as indicated by an increase in phosphorylated GSK3*β* (Figure 4(a)). As GSK3*β* was shown to inhibit the degradation process of *β*-catenin, we found corresponding results that phosphorylated GSK3*β* leads to an increase of cellular *β*-catenin in CIP treated DPCs (Figure 4(a)). These data suggested that CIP maintains stemness in DPCs at least in part by increased cellular *β*-catenin via Akt/GSK3*β* pathway.

Recently, the process of the cell transition from epithelial-mesenchymal phenotypes (EMT) has garnered increased attention in cell biology as it is linked with the stem cell-like properties in various cells [16–18]. Furthermore, the transcription factors upregulated during EMT like Snail were shown to maintain the stem cell-like phenotypes in many cells [16–18]. In order to clarify whether this EMT plays a part in stem cell maintenance of CIP, the EMT-activating transcription factors including ZEB1, Slug, and Snail were determined in the CIP treated cells by western blotting. After incubation with CIP for 72 h, the cellular levels of ZEB1 and Snail were significantly upregulated; however, we found only minimal change in case of Slug (Figure 4(b)). Moreover, we have determined the levels of downstream gene targets of Snail including N-cadherin and vimentin [28, 29]. The results indicated that such proteins are significantly upregulated in the CIP-treated cells (Figure 4(c)). It is worth noting herein that p-Erk (Thr 202/Tyr 204), an activation downstream target of Snail [30], was also found to be increased in CIP-treated cells. Taken together, our results revealed the novel molecular mechanism by which CIP mediates the stem cell-like phenotypes in the DPCs through *β*-catenin and EMT.

### 3.5. CIP Increases the Self-Renewal Transcription Factors in DPCs

Self-renewal is an important signature of stem cells [31–33]. To provide supportive data on the effects of CIP on DPCs stemness, critical transcription factors that maintain pluripotency and self-renewal in stem cells, including Nanog and Oct-4, were determined [34, 35]. After treatment of the cells with 5 and 10 *μ*g/mL of CIP (Figure 4(b)), the cells exhibited dramatic increases of Nanog and Oct-4 in a dose-dependent manner, suggesting that the treatments induced the self-renewal machinery in these cells.

### 3.6. CIP Maintains the Stem Cell-Like Phenotypes of Primary Human DPCs

**Figure 5:**
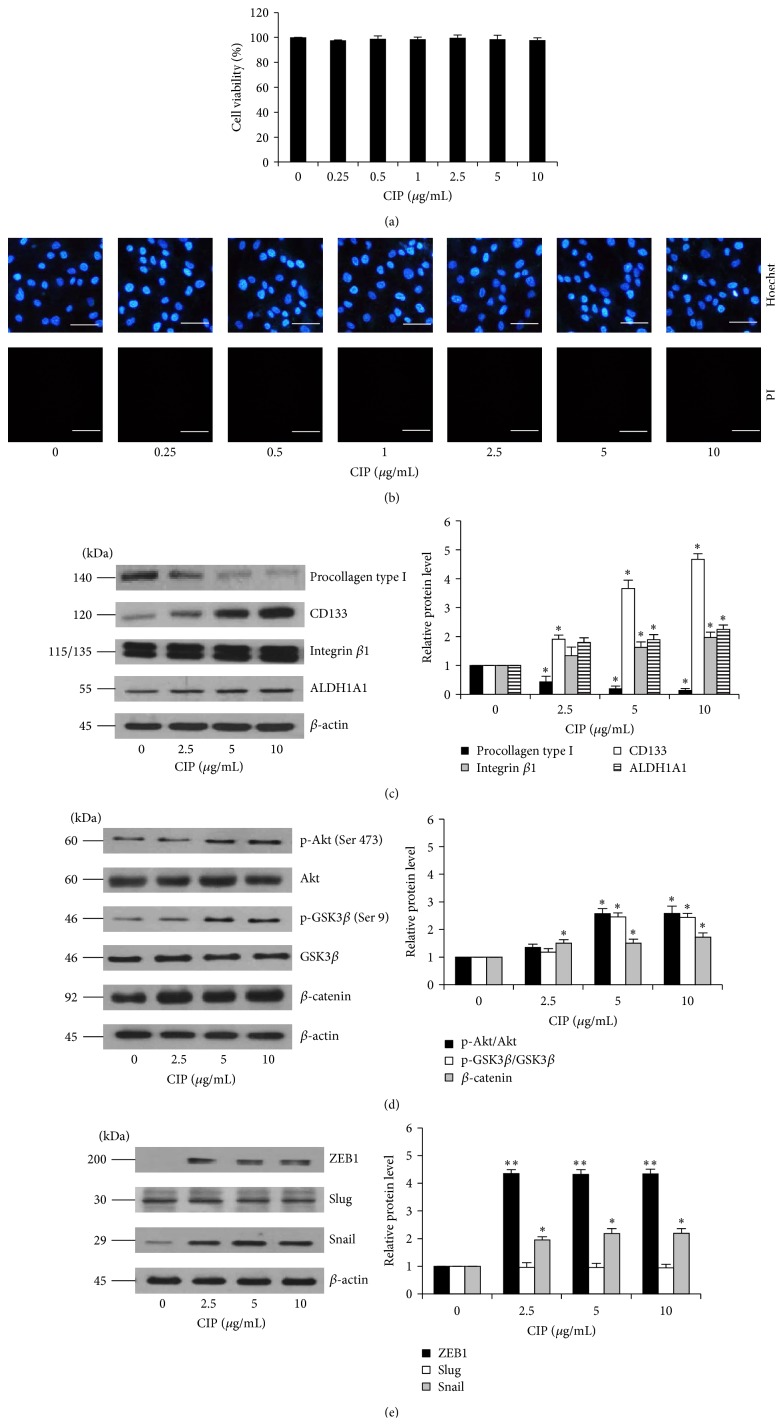
Effects of CIP on stem cell-like phenotypes in primary human DPCs. (a) Cells were treated with CIP (0–10 *μ*g/mL) for 24 h. Cytotoxicity was determined by MTT assay. The data represent the means of four independent triplicate samples ± SD. (b) After indicated treatment, mode of cell death was examined by Hoechst 33342/PI costaining assay. Scale bar is 100 *μ*m. ((c)–(e)) Cells were treated with CIP (0–10 *μ*g/mL) for 72 h. After indicated treatment, the levels of stem cell markers (procollagen type I, CD133, integrin *β*1, and ALDH1A1), Wnt/*β*-catenin signaling (Akt, p-Akt, GSK3*β*, p-GSK3*β*, and *β*-catenin), and EMT transcription factors (ZEB1, Slug, and Snail) were determined by western blot analysis, respectively. *β*-actin was served as the loading control. The immunoblot signals were quantified by densitometry and mean data from independent experiments were normalized to the results. The data represent the means of four independent samples ± SD. ^*∗*^ *P* < 0.05 and ^*∗∗*^ *P* < 0.01 versus untreated control.

To determine the extent to which primary human DPCs will respond to the CIP treatment by the same manner, we treated the isolated human DPCs with 0–10 *μ*g/mL CIP and evaluated stem cell characteristics, accordingly. Figures 5(a) and 5(b) indicate that treatment of the cells with 0–10 *μ*g/mL CIP caused no direct cytotoxicity in these cells. We next tested the signature characteristics of stem cells to assess whether CIP sustained the stemness in these primary cells. The cells were cultured in the presence or absence of CIP for 72 h and the expression of stem cells as well as fibroblast markers was determined as described previously. Western blot analysis revealed that the levels of CD133, integrin *β*1, and ALDH1A1 were significantly increased in response to CIP treatment in a dose-dependent manner, whereas the expression of procollagen type I was significantly suppressed (Figure 5(c)). Furthermore, the Wnt/*β*-catenin and EMT were evaluated by western blotting.  Figure 5(d) shows that treatment of the cells with CIP significantly increased the level of activated Akt level, inactivated GSK3*β*, and *β*-catenin. Besides, the EMT transcription factors ZEB1 and Snail were found to be upregulated in response to the treatment (Figure 5(e)). Taken together, these data supported our earlier findings that CIP maintains the stemness of DPCs via *β*-catenin and EMT.

## 4. Discussion

DPCs have been shown to exhibit phenotypic plasticity by differentiating to different cell types [36]. Interestingly, multipotency of DPCs is accepted to be an important factor determining an ability to induce hair follicle formation [10–12]. Although advancement in research facilitates the culture of these specialized cells, previous study has shown that the hair inducing property of DPCs is gradually declined during culture [4, 6, 7]. In an attempt to evaluate the possible ways to maintain the DPCs signature* in vitro*, we have discovered for the first time that CIP was able to prevent the loss of DPCs stemness during culture. Treatment of the DPCs with nontoxic concentration of CIP prevented the spontaneous alteration of cell morphology toward fibroblast-like cells (Figure 2(a)). Also, our immunocytochemistry as well as protein analysis revealed that the cultured DPCs decreased the stem cell markers, and that could be prevented by an addition of CIP (Figures 3(a) and 3(b)). In addition, we found the increase of procollagen type I in the DPCs, suggesting that the DPCs can instinctively differentiate to fibroblast-like cells (Figure 3(b)). This observation is in agreement with the previous reports indicating that DPCs differentiate toward fibroblast-like cells [23, 24].

Regarding stem cell research, CD133, a transmembrane glycoprotein, has been widely used as a standard biomarker of stem cells [37]. We found that DPCs at the early passages exhibited high level of CD133; however, the expression of this protein was found to decline in a time-dependent manner during the time of cultivation, indicating that the cells have lost their stemness. Together with other stem cell markers, CIP was shown to sustain stemness of DPCs as indicated by the steady level of CD133, ALDH1A, and integrin *β*1 (Figure 3(b)). Besides, the presence of ALDH1A1, a detoxifying enzyme highly expressed in stem cells, was shown to regulate stem cell function [38]. Thus the increase of the proteins in response to CIP treatment could support our finding that CIP maintains the stemness of the cells.

In the past decade, considerable progress has been obtained in elucidating stem cell signaling pathways, in particular Wnt/*β*-catenin that is critical for maintaining stem cell features as well as function [25–27]. Previously, studies reported that an ablation of *β*-catenin in DPCs causes the suppression of hair growth and regeneration [13]. The *β*-catenin functions as a cotranscription factor of T-cell factor/lymphoid enhancing factor (TCF/LEF) and consequently regulates expression of proteins facilitating stem cell functions [39]. The cellular level of *β*-catenin is tightly controlled by GSK3*β*. The phosphorylation of *β*-catenin by the function of GSK3*β* resulted in ubiquitination and proteasomal degradation of *β*-catenin. The activated Akt is shown to inhibit such a function of GSK3*β* by phosphorylating the GSK3*β* at serine 9 [39]. Therefore, the activation of Akt increases cellular level of *β*-catenin. As a consequence, *β*-catenin accumulates in cytoplasm and translocates into nucleus leading to stimulation of target genes. Here, we showed that the levels of activated Akt and inactivated GSK3*β* were upregulated consistently with the increase of *β*-catenin in CIP treated cells (Figures 4(a) and 5(d)). These results suggested that the stemness sustaining effect of CIP is due to the activation of Akt/*β*-catenin pathway.

Indeed, the DPCs require stemness in terms of molecular signals rather than pluripotency for the production of cytokines and growth factors functioning in the keratinocyte recruitment and proliferation. In this regard, activation of Wnt/*β*-catenin signaling was shown to induce hair follicle formation and hair growth through stem cell signals that drive cytokine synthesis [7, 13, 40, 41]. For the focus points of this study, the upregulation of stemness signals including *β*-catenin, Nanog, Oct4, Slug, and Snail described the mechanism of ciprofloxacin in driving the DPCs to functioning in enhancing the growth of hair (Figures 4 and 5).

Recent evidences have suggested that *β*-catenin interacts with many signaling pathways involved in pluripotency and EMT [25–27, 35, 42]. Furthermore, Wnt/*β*-catenin signaling activation was shown to increase the expression of EMT proteins and pluripotent activating transcription factors [43–47]. Consistent with previous reports that the transcription factors Snail and ZEB1 play an important role in EMT process [16, 18], we found significant increase of such proteins in the DPCs treated with CIP (Figures 4(b) and 5(e)), and the increase of EMT markers was found to be corresponding to the stem cell-like morphology and aggregative behavior. Our findings also lend strong support to the view that Akt/GSK3*β*-dependent *β*-catenin upregulation is important for the DPCs to maintain their stemness. We unveiled that the transcription factors which are downstream targets of Wnt/*β*-catenin, namely, Nanog and Oct4, were upregulated in the CIP-treated cells.

**Figure 6:**
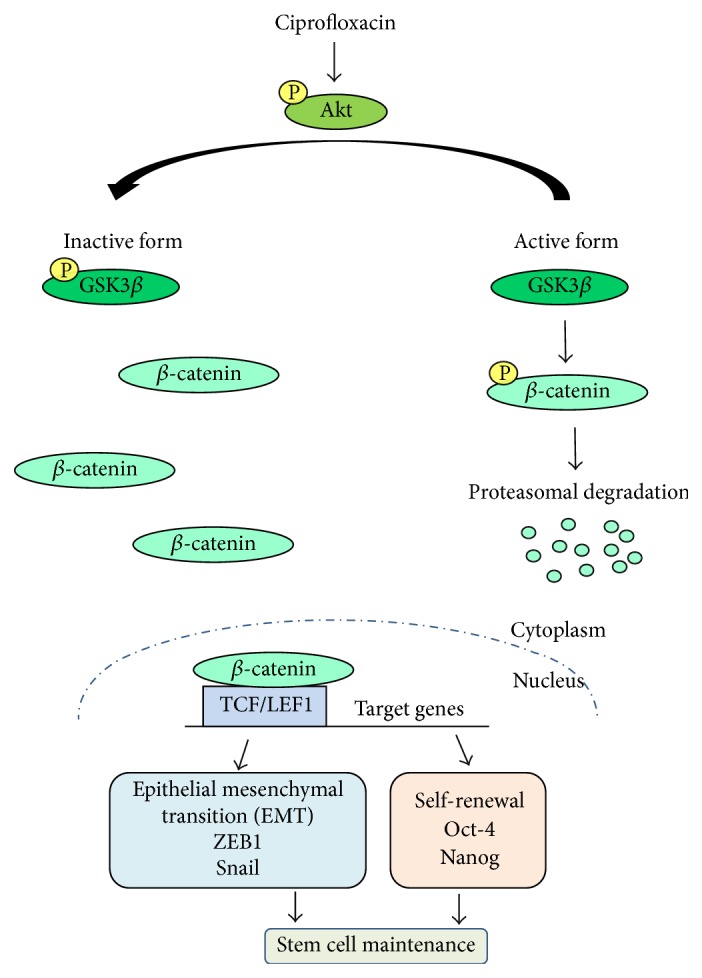
Schematic diagram summarizes the effects of CIP for improvement of the stemness in human DPCs. CIP improved the stemness of human DPCs through Akt activation which accounts for GSK3*β* inactivation, resulting in the increase of cellular *β*-catenin. As a consequence, *β*-catenin accumulates in cytoplasm and translocates into nucleus leading to stimulation of target genes, including transcription factors associated with EMT and self-renewal that might exert the stemness sustaining effect of CIP.

In closing, we systemically evaluated the positive role of CIP treatment for the maintenance of stemness in cultured DPCs. We identified a novel finding on the stemness regulatory effect of CIP in DPCs, that is, through Akt/GSK3*β*-dependent *β*-catenin signal resulting in an upregulation of transcription factors associated with EMT and self-renewal (Figure 6). This information may open the door to further investigations and make this new application of the drug in culture be useful for the cell therapeutic approaches.

## Abbreviations

DPCs: Dermal papilla cells
GSK3*β*: Glycogen synthase kinase3*β*
Akt: ATP-dependent tyrosine kinase
EMT: Epithelial-mesenchymal transition
FGF: Fibroblast growth factor
IGF: Insulin-like growth factor
CIP: Ciprofloxacin
TCF: T-cell factor
LEF: Lymphoid enhancing factor.
